# Eating disorders in medicine university students in a city in the interior of the state of São Paulo Brazil

**DOI:** 10.1192/j.eurpsy.2024.488

**Published:** 2024-08-27

**Authors:** M. C. V. R. De Oliveira, C. B. Soares de Oliveira, N. D. Rejali, M. B. Machado, M. H. Formaio, S. Umbelino da Silva

**Affiliations:** FACULDADE DE MEDICINA, UNOESTE, PRESIDENTE PRUDENTE, Brazil

## Abstract

**Introduction:**

Eating disorders are characterized by a persistent disturbance in eating and/or eating-related behavior, resulting in altered food consumption or absorption, which can significantly compromise physical health as well as psychosocial functioning. These disorders are closely linked with stressful experiences which university students configure a group prone to development.

**Objectives:**

The objective is to evaluate the impact of eating disorders on young people when entering and staying at university.

**Methods:**

This is an observational, quantitative, analytical and cross-sectional study, in which 1300 (one thousand and three hundred) medical students were invited, of both sexes and over 18 years of age from the 1st (first) to the 12th (twelfth) year. period of the Medicine course at the University of Oeste Paulista (UNOESTE) with 91 students joining. A structured interview was applied via online, aiming at collecting sociodemographic and occupational data in conjunction with the application of the Periodic Eating Compulsion Scale - ECAP, assessing the existence and degree of eating disorders in medical students.

**Results:**

Mean age 22.7 ± 3.9 years, predominantly female (76.9%) and white ethnicity (86.8%). Most live alone or with a parent (82.5%). With regard to eating habits, 81 (89.0%) said they did not follow a nutrition professional’s diet, and 84 (92.3%) have at least 3 meals a day. Lunch is eaten by 100% of the participants, while supper is the least consumed meal (17.6%). A total of 24 (26.4%) participants said they had little time to eat, and almost half (46.2%) did not prepare their own meals, with 12.5% choosing to eat salted or not. eating a certain meal. The ECAP binge eating score had a median of 9 (11.5) points, with a minimum score equal to 1 and a maximum equal to 41. Sixty-eight (74.7%) of the participants were classified as having no binge eating, with moderate binge eating 15 (16.5%), and severe, 8 (8.8%).

**Conclusions:**

There is a need for changes in lifestyle aspects in order to present healthier meals in appropriate amounts, in addition to an adequate therapeutic approach to these disorders. Research funding agency We also declare that we received financial support from the Institutional Program for Scientific Initiation Scholarships (PROBIC).

**Disclosure of Interest:**

None Declared

